# Cardiovascular effects on high-resolution 3D multi-shot diffusion MRI of the rhesus macaque brain

**DOI:** 10.1162/imag_a_00039

**Published:** 2023-12-08

**Authors:** Yann Bihan-Poudec, Slimane Tounekti, Thomas Troalen, Holly Rayson, Mathilda Froesel, Franck Lamberton, Zakaria Zariry, Maëva Gacoin, Nathalie Richard, Suliann Ben Hamed, Bassem Hiba

**Affiliations:** Institut des Sciences Cognitives Marc Jeannerod, CNRS UMR 5229, Lyon, France; Université Claude Bernard Lyon I, Villeurbanne, France; Department of Radiology, Thomas Jefferson University, Philadelphia, Pennsylvania, United States; Siemens Healthcare SAS, Avenue des Fruitiers, Saint-Denis, France; Cermep, Imagerie du Vivant Lyon, Lyon, France; SFR Santé Lyon-Est, CNRS UMS3453, INSERM US7, Université Lyon 1, Lyon, France

**Keywords:** high-resolution brain imaging, macaque brain, diffusion MRI, triggered diffusion MRI, 3D multi-shot EPI, cardiovascular effect

## Abstract

The monkey brain represents a key research model thanks to its strong homologies with the humans, but diffusion-MRI (dMRI) performed at millimeter-level resolution using clinical scanners and pulse-sequences cannot take full advantage of this. Cardiovascular effects on 3D multi-shot Echo-Planar Imaging (3D-msEPI) dMRI were characterized at submillimetric resolution by comparing triggered and non-triggered diffusion-weighted (DW)-images and diffusion tensor imaging (DTI) maps. We also investigated the value of 3D-msEPI with cardiovascular-triggering to achieve dMRI of the anesthetized macaque brain with high resolution previously restricted to *ex-vivo* brains. Eight DW-images with voxel-size = 0.5 × 0.5 × 1 mm^3^ and b = 1500 s/mm^2^ were collected at 3 Tesla from two macaques using triggered and then non-triggered 3D-msEPI. Statistical analysis by mixed models was used to compare signal-to-noise ratio (SNR) and ghost-to-signal ratio (GSR) of DW-images with and without triggering. Brain DTI with isotropic-resolution of 0.4 mm and b = 1000 s/mm^2^ was also collected in three macaques with triggered 3D-msEPI and reapplied without triggering in one. Cardiovascular pulsations induce inter-shot phase-errors with non-linear spatial dependency on DW-images, resulting in ghost-artifacts and signal loss particularly in the brainstem, thalamus, and cerebellum. Cardiovascular-triggering proved effective in addressing these, recovering SNR in white and gray matter (all p < 0.0001), and reducing GSR from 16.5 ± 10% to 4.7 ± 4.2% on DW-images (p < 0.0001). Triggered 3D-msEPI provided DTI-maps with the unprecedented spatial-resolution of 0.4 mm, enabling several substructures of the macaque brain to be discerned and thus analyzed *in vivo*. The value of cardiovascular-triggering in maintaining DTI-map sharpness and guaranteeing accurate tractography results in the brainstem, thalamus, and cerebellum was also demonstrated. In conclusion, this work highlights the effects of cardiovascular pulsations on brain 3D-dMRI and the value of triggered 3D-msEPI to provide high-quality diffusion-MRI of the anesthetized macaque brain. For routine studies, 3D-msEPI must be coupled with appropriate techniques to reduce acquisition duration.

## 1 Introduction

Diffusion magnetic resonance imaging (dMRI) is a unique tool for probing the microstructure and the connectivity of living cerebral tissues in normal and pathological conditions. Several reports have highlighted the benefits of high-resolution dMRI to characterize small anatomical structures of the brain (Crombe et al., 2018; Zhang et al., 2021), to enhance white-matter tractography (Kamali et al., 2014; Richard et al., 2021; Steele et al., 2016), and also to define new prognostic biomarkers of pathological, pharmacological, or physiological conditions (Griton et al., 2020; Planche et al., 2017).

When high-resolution dMRI is needed, the readout duration of echo-train collected using the standard 2D single-shot echo-planar-imaging (2D-ssEPI) becomes too long. This makes ssEPI very vulnerable to several sources of artifacts (T2* decay, geometric distortions, susceptibility, and eddy currents) and limits its ability to achieve high-resolution images. To avoid these undesirable effects, high-resolution dMRI can be performed using a multi-shot EPI (msEPI). In each shot, a fraction of the Fourier-space is recorded with a phase-error, induced by the head rigid-motions or by cardiovascular pulsations occurring between spins excitation and echo-train acquisition (Anderson & Gore, 1994; Atkinson et al., 2000). Additional inter-shot phase-errors are accumulated when 3D sampling of Fourier-space is employed in order to enhance the signal-to-noise ratio (SNR) efficiency (Chang et al., 2018; Engström & Skare, 2013; Van et al., 2011). The large diffusion-weighting gradient pulses, which sensitize magnetic-resonance (MR)-signal to the microscopic diffusion of water spins, amplify inter-shot phase-errors induced by the head rigid-motions and by the cardiovascular pulsations, and thus exacerbates aliasing artifact and/or signal loss when 3D-msEPI is used to collect dMRI data. 3D-msEPI must, therefore, always be coupled with an appropriate inter-shot phase-errors correction strategy in order to obtain artifact-free diffusion-weighted (DW)-images (Chang et al., 2018; Engström & Skare, 2013).

The main aim of this study was to accurately characterize inter-shot phase-errors induced by the cardiovascular pulsations on anesthetized macaque brain DW-images collected at high spatial-resolution and with a standard diffusion-weighting (b = 1000 to 1500 s/mm^2^) using 3D-msEPI. Imaging anesthetized Rhesus macaques, with the head carefully restrained in a stereotaxic frame, provides the advantage of significantly reducing rigid bulk motion. We can thus focus on the characterization and the compensation of the physiological motion effects on 3D-msEPI dMRI. The effects of cardiovascular-pulsations were analyzed by comparing triggered and non-triggered DW-images.

This study investigates also the benefits of 3D-msEPI acquisitions with cardiovascular triggering to achieve high-quality dMRI data of the anesthetized macaque brain with a spatial-resolution similar to those only achievable post-mortem. Indeed, in order to take full advantage of dMRI of the monkey brain, which has very strong functional and structural homologies with the human brain (Milham et al., 2018), several *ex-vivo* studies have reported very high-resolution dMRI of the fixed macaque brain (Supplementary Data S1). While post-mortem dMRI has brought important advances to the field, it does not maintain the non-invasive aspect of dMRI and excludes a wide range of longitudinal non-human primate studies in pharmacology and neurosciences. In addition, water diffusivity and core physiological properties of gray and white matter change as a result of brain fixation of *ex-vivo* biological tissues (D’Arceuil & De Crespigny, 2007).

Only a few reports have described cerebral dMRI in anesthetized macaque at a sub-millimetric cubic resolution. Janssens et al. (2012) developed a high-sensitivity 8-channel phased array coil with loops implanted directly onto the skull of a macaque monkey. Using these invasive coils, diffusion tensor imaging (DTI) was collected at an isotropic spatial-resolution of 0.7 mm. Tounekti et al. (2018) have used a 3D-msEPI pulse sequence to non-invasively achieve DTI maps with a spatial-resolution of 0.5 mm of anesthetized macaque brains, and have also reported a degree of vulnerability of this sequence to the effects of cardiac pulsatility. In a recent study by Grier et al. (2022), a 10.5 Tesla ultra-high-field MRI scanner was used to perform 2D-ssEPI dMRI of the anesthetized macaque brain at the spatial-resolution of 0.58 mm. In the current study, cerebral DTI with an isotropic spatial-resolution of up to 0.4 mm (voxel-volume of 0.064 mm^3^) was collected in anesthetized monkeys using triggered 3D-msEPI pulse sequence and a standard 3 Tesla MRI scanner.

## 2 Methods

This study was authorized by the French Ministry for Higher Education and Research (project no. 2016120910476056), in accordance with the French transposition texts of Directive 2010/63/EU and in compliance with the ARRIVE guidelines. Four Rhesus monkeys were included in this study (M1 is a 16-year-old female weighing 7.02 kg; while M2, M3, and M4 are 14-, 12-, and 7 year-old males weighing 7.20, 9.08, and 9.15 kg respectively). For each experiment, the subject was first injected with an intramuscular glyocopyrrolate (Robinul-V, Vetoquinol, 5 mg/kg), and 10 minutes later, with ketamine (Ketamine, 1000, Virbac, 10 mg/kg) to induce anesthesia. The monkey was then intubated and maintained with isoflurane at a concentration between 1.5 and 1.75%, and at a respiratory frequency of 20 resp/min. Electrocardiogram (ECG) electrodes were placed on the subject’s thoracic cage and a pulse oximeter on their toe. The subject was finally placed in a sphinx position within a stereotaxic frame. Physiological parameters (temperature and respiratory, heart, blood oxygenation, and expired CO_2_ rates) were monitored. Heart rate was kept stable throughout the dMRI scan by adjusting the isoflurane concentration, respiratory volume, and temperature of a warming blanket.

dMRI scans were performed on a 3T Magnetom Prisma system (Siemens Healthineers, Germany). The whole-body coil was used for the radio frequency (RF) transmission and three Siemens surface coils for MR-signal reception. Two 11 cm diameter loop coils were placed on both sides of the head and a 7 cm diameter loop coil on the top of the head.

### 2.1 Triggered 3D-msEPI dMRI

A 3D-msEPI dMRI pulse sequence was used in this study. EPI readouts were used to scan the *k*-space in *k_xy_*. MR-signal collected from the slab was phase-encoded along *k_z_* to generate a 3D *k*-space. The Stejskal-Tanner preparation scheme was then introduced to create diffusion-weighting (Stejskal & Tanner, 1965). To address fat-related artifacts, a binomial pulse was used to selectively excite water protons only, prior to diffusion encoding (Hauger et al., 2002). Slab-selective 90° (3.44 ms binomial pulses, bandwidth-time product of 5.2) and 180° (3.3 ms Sinc pulse) RF pulses were used respectively for excitation and refocusing of spins inside the slab. To reduce the echo-train duration (ETD) and echo-time (TE), each *k_z_* plane (*k_xy_*-space) was sampled through numerous interleaved segments, each sampled with one EPI-readout (Tounekti et al., 2018). Partial-Fourier was used in *k_y_* to reduce the ETD and the TE, and in *k_z_* to shorten acquisition time. The *k*-space orientations *k_x_*, *k_y_*, and *k_z_* were set respectively to left-right, posterior-anterior, and inferior-superior directions based on the brain frame. Tables 1 and 2 summarize the dMRI pulse sequence parameters for each acquisition in the study.

**Table 1.** dMRI pulse sequence parameters for each acquisition.

dMRI acquisitions	Resolution (mm^3^)	Matrix size (pixels)	b-Value (s/mm²)	# of shots	TR (R-R)	TE (ms)	*k_y_* partial Fourier	*k_z_* partial Fourier	# of subjects
Acq. 1	0.5 × 0.5 × 1.0	250 × 212 × 112	1500	3	2	74	0.75	0.75	2
Acq. 2	0.4 × 0.4 × 0.4	304 × 254 × 128	1000	4	2	82	0.63	0.75	3

**Table 2.** Summary of all the acquisitions performed in this study.

dMRI acquisitions	Subject	Isoflurane concentration (%)	Trig	Cardiac rate (bpm)	TR (ms)	Total acquisition time
Acq. 1	M1	1.55	No	111 ± 0.5	1000	17 min
	M1	1.55	Yes	112 ± 0.7	2 × RR	17 min 2 s
	M4	1.5	No	118 ± 0.5	1000	17 min
	M4	1.5	Yes	119 ± 1.6	2 × RR	17 min 7 s
Acq. 2	M1	1.55-1.7	Yes	111 ± 1.1	2 × RR	1 h 53 min 54 s
	M2	1.5-1.75	Yes	114 ± 2.0	2 × RR	1 h 57 min 45 s
	M3	1.5-2	No	109 ± 2.3	1200	2 h 6 min 57 s
	M3	1.5-1.75	Yes	115 ± 1.7	2 × RR	1 h 58 min 3 s

The mean ± SD of the heart rate are presented for each acquisition.

The 3D-msEPI dMRI pulse sequence was used in triggered mode, such that Fourier-space partition sampling was time-locked to a specific period of the cardiac cycle. Since the ECG-signal is very vulnerable to electromagnetic interference, the peripheral-pulse oximeter was used for the triggering of dMRI-acquisition. The ECG-signal was only used before each scan to estimate the delay required to achieve peripheral triggering. A trigger-delay of 70% of the interval elapsed between two successive R-waves was used for all the triggered acquisition in this study (See Supplementary Data S2). dMRI-acquisition was triggered once every two cardiac cycles.

### 2.2 Diffusion-MRI acquisition

The effects of cardiovascular pulsation on each individual DW-image depend on its b-value and on its diffusion encoding direction, and the sum of these effects on all DW-images together will impact the DTI maps quality. We have therefore collected two sets of dMRI-acquisition for two complementary purposes:

#### 2.2.1 Investigate the effects of cardiovascular pulsations on DW-images (Acq. 1)

The aim of this first experiment was to characterize the intershot phase-errors induced by blood and cerebrospinal fluid (CSF) pulsations on DW-images. Eight triggered and eight non-triggered DW-images were collected using 3D-msEPI in two macaques (Acq. 1 of Tables 1 and 2). A relatively high diffusion-weighting (b = 1500 s/mm^2^) was applied to encode the water diffusivity along the inferior-superior orientation of the macaque brain. This specific direction was chosen because it produces a high level of ghost-artifacts and signal loss (Nunes et al., 2005).

#### 2.2.2 Investigate the value of triggered 3D-msEPI for high-resolution DTI maps (Acq. 2)

Triggered DTI maps with a spatial-resolution of 0.4 mm were achieved in three monkeys (M1, M2, and M3). For each DTI dataset, 21 axial DW-images were acquired with a b = 1000 s/mm^2^ applied along non-collinear diffusion-encoding vectors generated using an electrostatic repulsion model (Caruyer et al., 2013). Supplementary Data S3 contains the diffusion direction vectors used for these acquisitions. A b0-image, without diffusion-weighting, and a b0-image with a reversed phase-encoding direction (posterior-anterior) were also collected. The acquisition was repeated twice in M3 monkey, once without and once with cardiovascular-triggering. The acquisition and the physiological parameters are summarized in Tables 1 and 2 (Acq. 2).

### 2.3 Data-processing

DW-images were reconstructed using the Gadgetron library (Hansen & Sørensen, 2013) with a noise pre-whitening (Hansen & Kellman, 2015) and an iterative Cuppen/projection onto convex sets algorithm for partial-Fourier reconstruction before the inverse Fourier-transform (Haacke et al., 1991). Off-resonance and eddy-currents effects on DW-images of the 4 dMRI datasets from Acq. 2 were corrected using the Topup and Eddy tools from the FMRIB-Software Library (Andersson & Sotiropoulos, 2016; Andersson et al., 2003). The DTI-maps (fractional anisotropy (FA), mean diffusivity (MD), axial diffusivity (AD), and radial diffusivity (RD)) were computed with Mrtrix3 (Tournier et al., 2012). A denoising step was applied in the magnitude domain only to the 4 dMRI datasets from Acq. 2 before Topup and Eddy using the MRtrix3 dwidenoise command (Veraart et al., 2016). To assess the potential of high-resolution DTI for the visualization and exploration of fine brain substructures, we downsampled dMRI data (Acq. 2) from 0.4 mm to 0.8, 1, and 1.2 mm by applying cubic interpolation using MRIConverter (https://surfer.nmr.mgh.harvard.edu/fswiki/mri_convert).

Deterministic tractography of the medial cerebellum peduncle (MCP) and the inferior cerebellar peduncle (ICP) was performed using triggered and non-triggered DTI data of M3. The tractography process was performed with MRtrix3 (Tournier et al., 2007). The tractography approach is based on the definition in each voxel of the fiber orientation distribution (FOD) using spherical deconvolution (Tournier et al., 2004). A sphere was defined within the brainstem as seeding region to generate 100,000 streamlines. A step size of 0.2 mm, an angle of 25°, and an FA cutoff of 0.28 were used for each tractogram.

### 2.4 Data analyses and statistics

The SNR values of Acq. 1 were computed as the ratio between the mean intensity assessed in a region-of-interest (ROI) and the standard-deviation of the noise assessed in the image background (Dietrich et al., 2007). ROIs manually drawn on the right hemisphere, on the left hemisphere, or on the central part of the brain are denoted hereafter with the subscript “l,” “r,” and “c,” respectively. White-Matter (WM) ROIs were manually drawn in the splenium of the corpus-callosum (SCCl, SCCr, and SCCc), in the genu of the corpus-callosum (GCCc), in the anterior commissure (ACc), in the posterior thalamic radiation (PTRl and PTRr), and in the cingulum (CGl and CGr). Gray matter (GM) ROIs were drawn in the thalamus (THl and THr), in the supramarginal gyrus (SMGl and SMGr), in the precentral gyrus (PGl and PGr), in the central part of the superior temporal gyrus (STGl and STGr), in the inferior part of the superor temporal gyrus (iSTGl and iSTGr), and in the middle temporal gyrus (MTGl and MTGr). All the WM and GM SNR-ROIs were manually defined on an axial slice of a triggered DW-image with the aid of the Neuromaps atlas. As the head was held in the stereotaxic frame without any possible skull motion, the same ROIs were used for the non-triggered DW-images. Two additional ROIs were manually traced on the medial sagittal slice of the brain in order to quantify the SNRs for the brainstem and for the cerebellum (Supplementary Data S4).

The ghosting artifacts of Acq. 1 were also evaluated as the ghost-to-signal ratio (GSR) (Chang et al., 2018; Zhang et al., 2021), computed as:

$$\text{GSR}=s_{g}/S_{b}$$

where S_b_ and S_g_ are the mean values of signal assessed in two ROIs drawn in the splenium of the corpus-callosum and in its ghost, respectively. The signal ROI delineating the splenium of corpus callosum, which appears as a hyper signal, was manually outlined on the triggered DW-images with the aid of the Neuromaps atlas. The ghost’s rectangular ROI was drawn on the non-triggered DW-images outside the brain, on the axial slice where the ghost of the splenium of corpus callosum is most obvious (Supplementary Data S5).

Statistical analyses of the SNR and GSR values measured for the DW-images of Acq. 1 DW-images were performed using R (v4.1.1; https://www.R-project.org/) and the lme4 (v1.1.27; (Bates et al., 2014)) and emmeans (v1.5.3; (Searle et al., 1980)) packages. Separate linear mixed models, one for WM ROIs and one for GM ROIs, were run with SNR values as the outcome measure. Trigger condition (non-triggered/triggered), ROI, and their interaction were included as fixed effects, and subject-specific intercepts as a random effect. Separate linear mixed models, one for the cerebellum and one for the brainstem, were run with SNR values as the outcome measure. Trigger condition (non-triggered/triggered) was included as a fixed effect, and subject-specific intercepts as a random effect.

A linear mixed model was also run with GSR values as the outcome measure. Trigger condition (non-triggered/triggered) was included as a fixed effect, and subject-specific intercepts as a random effect. For the SNR analyses, *p*-values were obtained using Type III Wald X^2^ tests, and significant main effects of condition were followed up by planned pairwise comparisons of least square means. Pairwise comparisons were Tukey-corrected for multiple comparisons, and degrees of freedom were approximated using the Kenward-Rogers method. For the GSR analysis, *p*-values were obtained using Type II Wald X^2^ tests.

## 3 Results

**Fig. 1.**
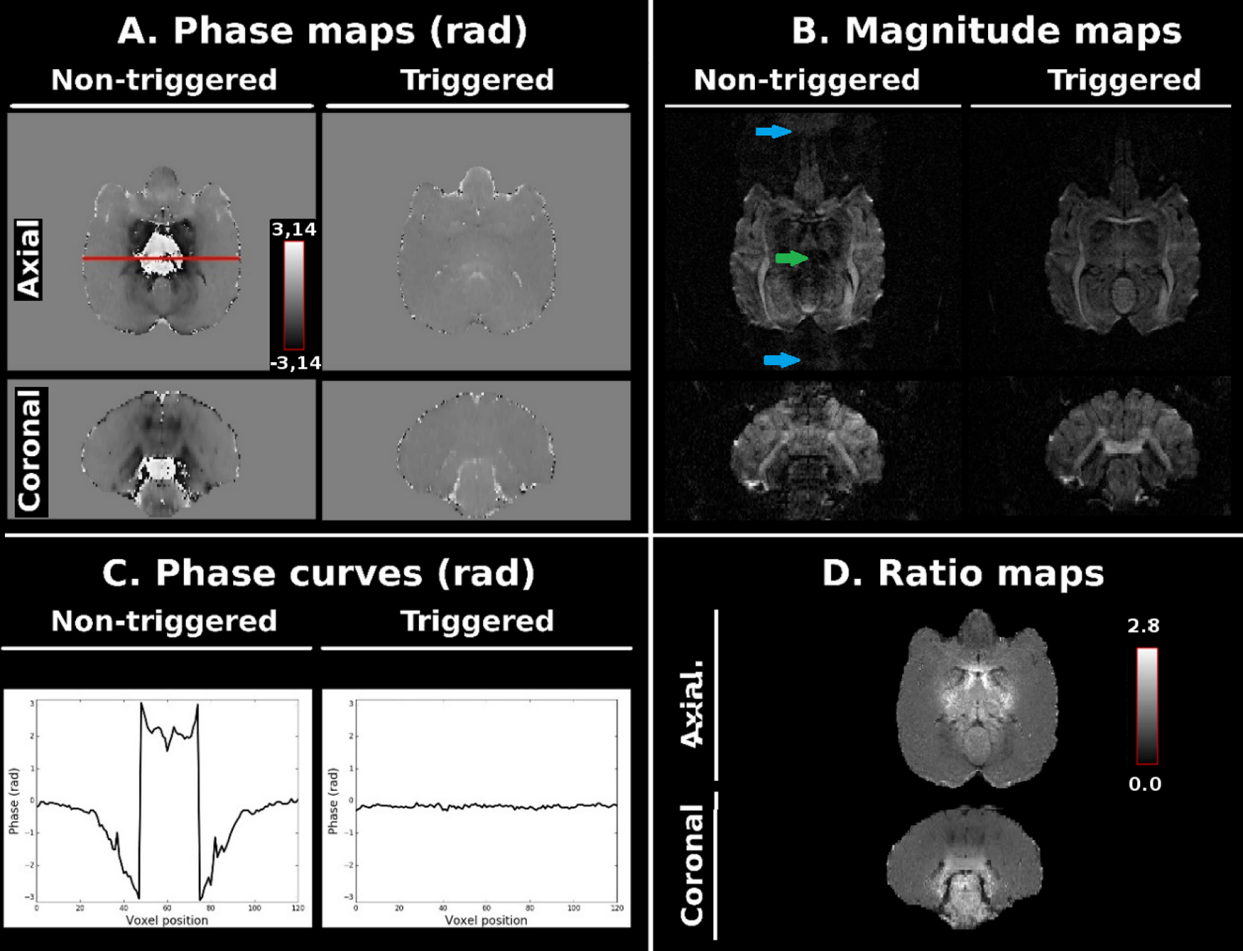
Coronal and axial views of phase-maps (A) and magnitude-maps (B) of the anesthetized macaque brain acquired with a b = 1500 s/mm² using 3D-msEPI. Non-triggered magnitude-maps suffer from significant signal loss (blue arrows) and ghosting artifacts (green arrow). The phase curves, corresponding to the red line in A, show the intershot phase-errors with nonlinear space dependence, induced by the cardiovascular pulsations only on the non-triggered images (C). The triggered to non-triggered magnitude ratio map illustrates the signal loss due to cardiovascular-pulsations (D).

Figure 1 illustrates the impact of cardiovascular-pulsations on macaque brain DW-images collected with a b = 1500 s/mm^2^ applied through the inferior-superior orientation. Cardiovascular pulsations corrupt DW-images by a phase-error which presents a nonlinear spatial distribution, mainly localized in brainstem, cerebellum, and thalamus regions (Fig. 1A and C, non-triggered phase-map). These phase-errors are negligible and have little to no effect on triggered phase-maps (Fig. 1A and C, triggered phase-map). Figure 1B shows that in comparison with triggered DW-images, non-triggered DW-images are corrupted by ghosting effects (green arrows) and also by signal dropout observed in brain regions suffering from nonlinear phase-errors (blue arrows).

The measured GSR values revealed that triggering the 3D-msEPI significantly decreased the ghost level (from 16.5 ± 10% for the 8 non-triggered DW-images to 4.7 ± 4.2%); a significant main effect of trigger condition [X^2^(1) = 16.68, p < 0.0001] was revealed, with GSR values lower in the triggered vs. non-triggered condition (See Supplementary Data S5).

Figure 1D shows axial and coronal views of the ratio map between the mean of the 8 triggered DW-images and the mean of the 8 non-triggered DW-images. This map illustrates the benefits of triggering in recovering the signal dropout introduced by cardiovascular-pulsations in the brainstem, cerebellum, and thalamus regions. The signal recovery provided by the cardiovascular trigger in two macaques is confirmed by the quantitative analysis results summarized in Supplementary Data S6 and S7.

For the WM SNR analysis (Supplementary Data S6), there were significant main effects of trigger condition [X^2^(1) = 122.77, p < 0.0001] and ROI [X^2^(11) = 1145.87, p < 0.0001], as well as their interaction [X^2^(11) = 166.1, p < 0.0001]. For the GM SNR analysis (Supplementary Data S7), significant main effects of trigger condition [X^2^(1) = 4.93, p = 0.03] and ROI [X^2^(8) = 224.49, p < 0.0001] were revealed, as well as their interaction [X^2^(8) = 56.84, p < 0.0001]. SNR was higher for triggered vs. non-triggered conditions for the SCCl, SCCr, SCCc, Acc, THl, and THr (see Table 3).

**Table 3.** Significant differences in SNR between triggered and non-triggered DW-images of the macaque brain (Acq. 1, 2 subjects, 8 triggered and 8 non-triggered DW-images each).

Brain matter	Region of interest	t	P
White matter	Splenium of corpus-callosum—left (SCCl)	t(269) = 9.39	<0.0001
White matter	Splenium of corpus-callosum—right (SCCr)	t(269) = 8.38	<0.0001
White matter	Splenium of corpus-callosum—center (SCCc)	t(269) = 9.34	<0.0001
White matter	Anterior commissure—center (ACC)	t(269) = 6.35	<0.0001
Gray matter	Thalamus—left (THl)	t(359) = 5.3	<0.0001
Gray matter	Thalamus—right (THr)	t(359) = 5.1	<0.0001

Cerebellum SNR measurements revealed a significant effect of the trigger condition [X^2^(1) = 35.88.08, p < 0.0001], with a higher SNR in the triggered condition compared to the non-triggered condition. There was also a significant effect of the trigger condition [X^2^(1) = 35.63, p < 0.0001] for the brainstem, with a higher SNR in the triggered condition compared to the non-triggered condition (Supplementary Data S4).

**Fig. 2.**
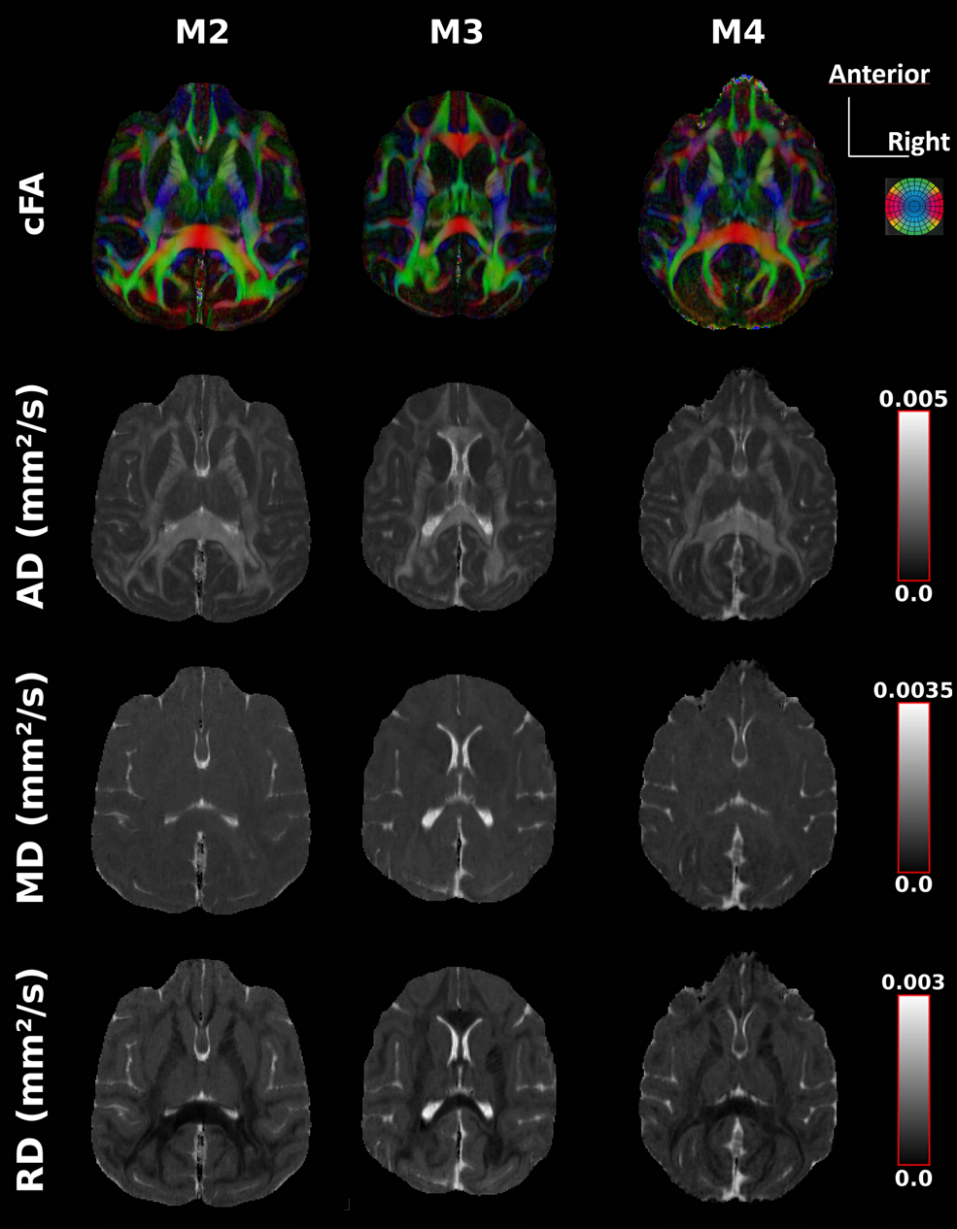
Axial views of diffusion tensor maps (colored fractional anisotropy (cFA), mean diffusivity (MD), axial diffusivity (AD), and radial diffusivity (RD)) of 3 anesthetized macaque brains achieved using triggered 3D multi-shot EPI at an isotropic spatial-resolution of 400 microns and with b = 1000 s/mm^2^. Color/gray scales are indicated on the right.

**Fig. 3.**
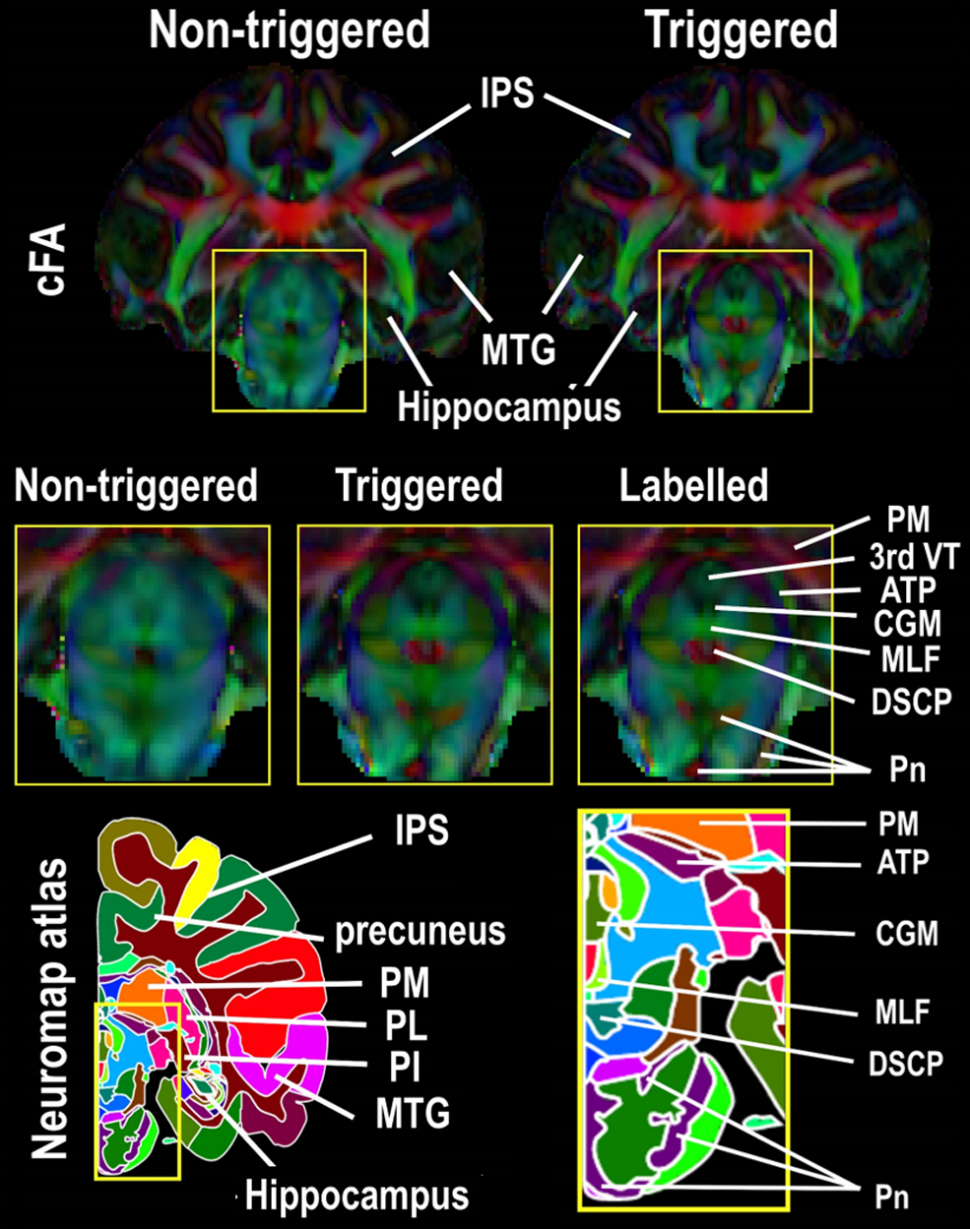
Coronal views of triggered and non-triggered colored fractional anisotropy (cFA) maps of the macaque brain with a resolution of 0.4 mm. The zoomed-in triggered cFA map reveals some substructures of the brainstem, such as central gray of the midbrain (CGM), medial longitudinal fasciculus (MLF), decussation of the superior cerebellar peduncle (DSCP), anterior pretectal nucleus (ATP), pontine nuclei (Pn), and third ventricle (3rd VT), which remain difficult to delineate on the non-triggered map. The intraparietal sulcus (IPS), middle temporal gyrus (MTG), medial pulvinar (PM), lateral pulvinar (PL), and inferior pulvinar (PI) are used as landmarks to locate the same brain sections on the cFA maps and on the Neuromap atlas.

**Fig. 4.**
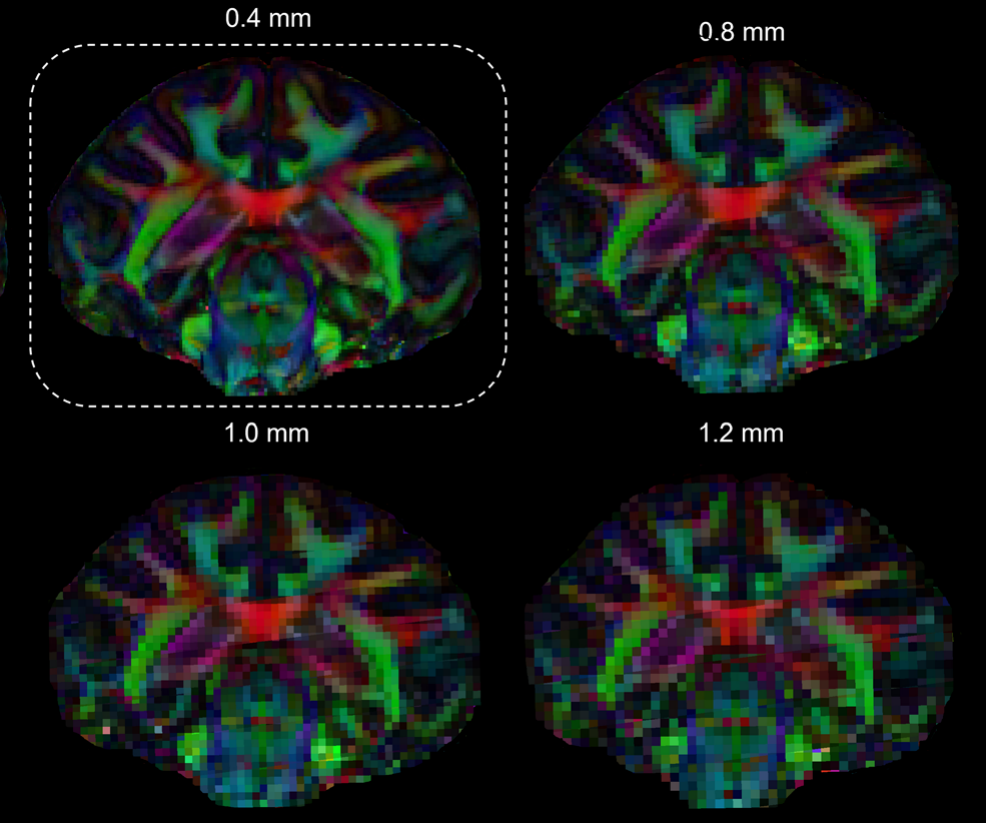
Coronal views of triggered colored fractional anisotropy (cFA) maps of the macaque brain achieved with a spatial-resolution of 0.4 mm, down-sampled up to 1.2 mm.

Figure 2 presents axial views of DTI-maps achieved with the spatial-resolution of 0.4 mm for three monkeys. The quality of these maps illustrates the feasibility of high-resolution DTI of the anesthetized macaque brain using triggered 3D-msEPI. The comparison between the triggered and non-triggered diffusion-tensor maps of M3 shows that the benefits of triggering are noticeable mainly in the brainstem, cerebellum, and thalamus regions affected by cardiovascular pulsations. For example, Figure 3 shows that the triggered color-coded fractional anisotropy (cFA) map reveals some substructures of the brainstem, such as the commissure of central gray of the midbrain, the medial longitudinal fasciculus, the decussation of the superior cerebellar peduncle, the anterior pretectal nucleus, the pontine nuclei, and the third ventricle, which remain difficult to delineate on the non-triggered cFA map. The brainstem sub-structures were defined according to the Neuromaps atlas (Rohlfing et al., 2012). The visual representation in Figure 4 clearly illustrates how these fine brainstem substructures are most clearly discernible at the 0.4 mm, lose their sharpness at the 0.8 mm resolution, and ultimately become indistinguishable at the standard resolutions of 1 to 1.2 mm.

**Fig. 5.**
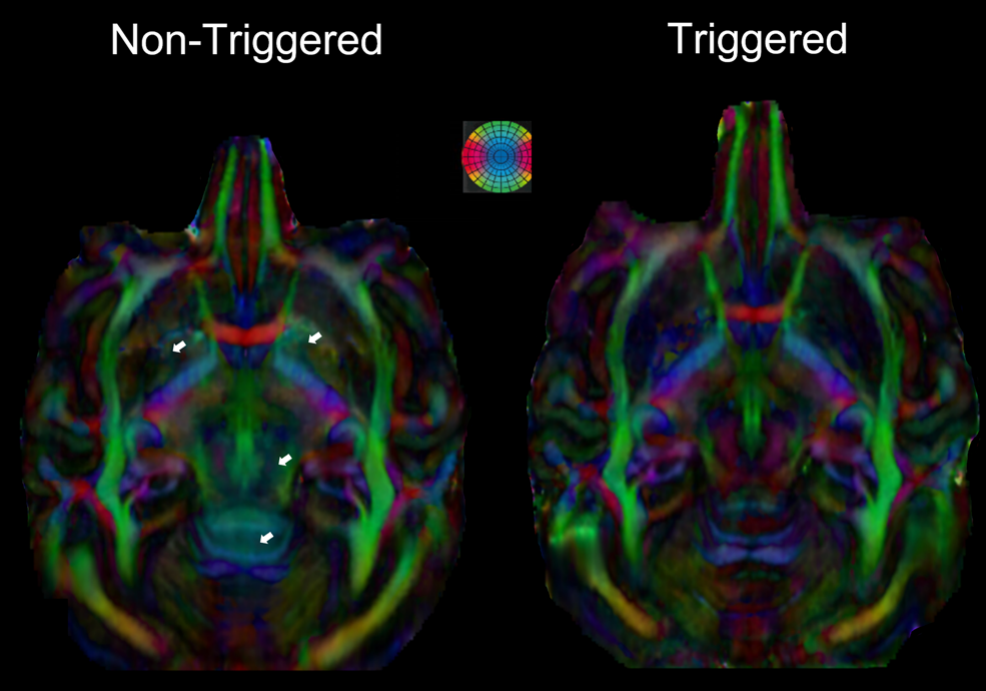
Axial views of triggered and non-triggered colored fractional anisotropy (cFA) maps of the macaque brain, achieved with a spatial-resolution of 0.4 mm. Arrows indicate artifacts induced by cardiac pulsations on the non-triggered FA map.

**Fig. 6.**
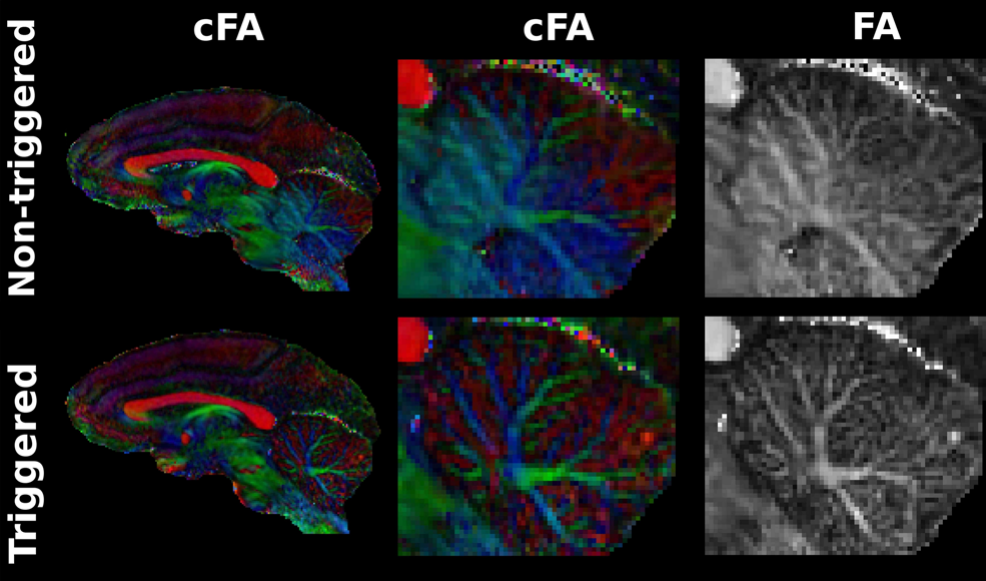
Sagittal views of triggered and non-triggered cFA (colored fractional anisotropy) maps of the macaque brain achieved with a spatial-resolution of 400 microns. A focus on the cerebellum reveals a blurring covering the complex anatomy of the cerebellum’s white matter when DTI maps are generated using non-triggered 3D multi-shot EPI.

Figure 5 shows the blurring generated by cardiac pulsations on the axial cFA map located in the thalamus, and the effectiveness of triggering in restoring the cFA sharpness. Similarly, cardiovascular triggering appears to be highly beneficial to recover and better delineate the cerebellum WM bundles. Visually, an abnormal anisotropy of water diffusivity is observable on non-triggered cFA map for a large anterior part of the cerebellum with a blue appearance (Fig. 6). This abnormal anisotropy, oriented along the brain in the inferior-superior direction, could be induced by tissue displacements occurring in the same direction during the cardiac cycle (Nunes et al., 2005).

**Fig. 7.**
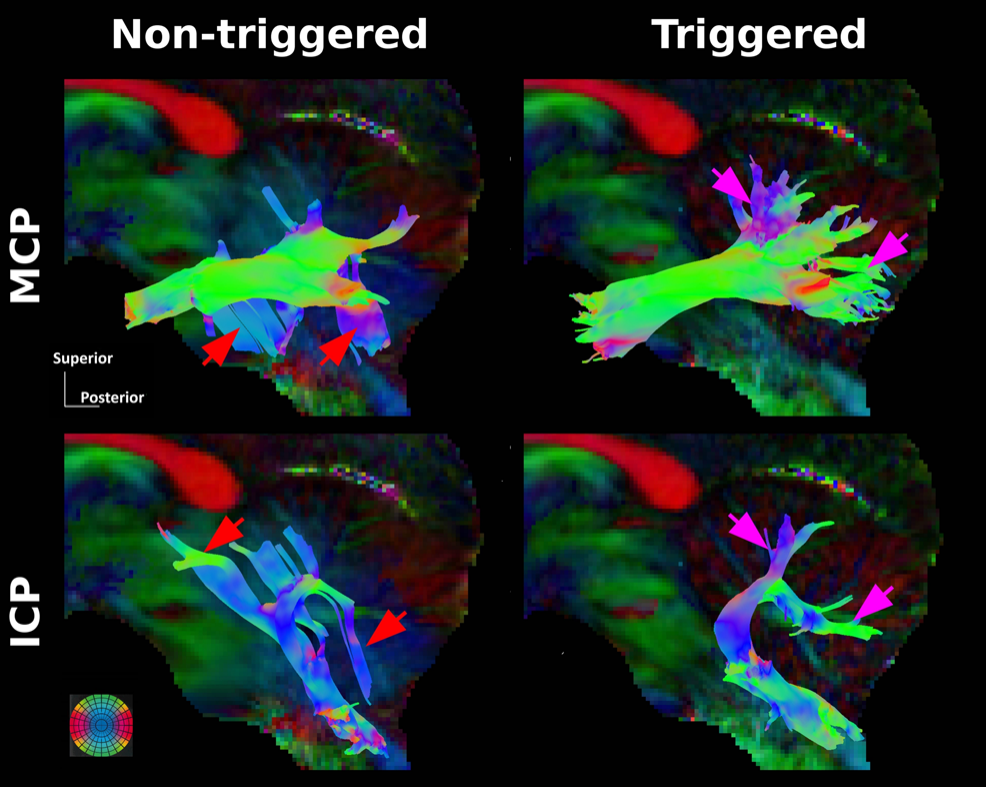
Tractograms of the macaque middle cerebellum peduncle (MCP) and inferior cerebellum peduncle (ICP). In comparison to the triggered tractograms, the red and the pink arrows indicate the false-positives and true-negatives generated by the non-triggered data, respectively.

The MCP and ICP tracks generated by tractography using the triggered dMRI data (Fig. 7) were in agreement with those obtained *ex vivo* by Calabrese et al. (2015). In the corresponding tracks obtained using non-triggered data, however, there are numerous false-positives (Fig. 7, red arrows) and true-negatives (Fig. 7, pink arrows).

### 3.2 Assessment of residual rigid movements of the macaque head held in a stereotaxic frame

We carried out additional investigations in 3 anesthetized macaques (M2, M3, and M4) in order to estimate the amplitude of residual rigid movements of the head restrained animals in a stereotaxic frame. For each monkey, 3 acquisitions were carried out to collect 197 sets of 3 slices in sagittal, coronal, and axial orientations, respectively, with an FOV = 160 × 102 mm^2^ (voxel-size = 1.7 × 1.7 × 2.5 mm^3^) using a 2D-ssEPI pulse sequence (TR/TE = 304/23.8 ms). The transformation matrices between successive images were computed among the 197 volumes using SPM (SPM12, http://www.fil.ion.ucl.ac.uk/spm) implemented in Matlab 9.1 (MathWorks, Inc., Natick, MA). These investigations confirm the negligibility of rigid movements (translations of 0.04 ± 0.03 mm, 0.07 ± 0.04 mm, and 0.13 ± 0.22 mm in left-right, posterior-anterior, and inferior-superior orientations respectively, and rotations of 0.05 ± 0.1°, 0.03 ± 0.007°, and 0.03 ± 0.003° around left-right, posterior-anterior, and inferior-superior axes respectively). These translation and rotation values were comparable to those measured using the 2D-EPI on an immobile phantom. The minor translation of the macaque brain in the inferior-superior orientation (0.13 ± 0.22 mm) could be induced by mechanic vibrations or by residual respiratory effects.

## 4 Discussion

3D-msEPI offers advantages such as reduced susceptibility to distortion and a better SNR to acquisition duration ratio (Engström et al., 2015; Tounekti et al., 2018), and therefore appears to be a promising alternative when high-resolution dMRI is required to investigate human and non-human brains (Crombe et al., 2018; Kamali et al., 2014; Planche et al., 2017). Tounekti et al. (2018) have demonstrated that 3D-msEPI of whole macaque brain improves the SNR efficiency of sub-millimeter dMRI by a factor of 4 to 5 when compared to 2D-ssEPI. Additionally, this technique has been shown to be useful for producing brain images with high-diffusion weighting, which can improve the sensitivity of dMRI to microstructural lesions in brain tissue (Crombé et al., 2022).

Correcting for motion artifacts in 3D-msEPI dMRI of the human brain is challenging due to the combined effects of random head motion and cardiovascular pulsations. 2D Navigator-echoes have been used to assess the inter-shot phase-errors and to correct their effects on the 3D-msEPI dMRI. However, the efficiency of this approach is valid only for the relatively thin slab (e.g., <30 mm), with the assumption that phase variations are negligible in the *k_z_* orientation (Engström & Skare, 2013). Chang et al. (2018) have demonstrated that motion-induced phase inconsistencies along the *k_z_* component could be corrected using 3D-navigator echoes in order to achieve near whole brain dMRI data using a single slab of 100 mm. In contrast, phase-errors in 3D-msEPI dMRI of anesthetized macaque brains held in a stereotactic frame are mainly induced by cardiovascular pulsatility and are less affected by rigid head movements. Consequently, such phase-errors can be successfully addressed by implementing cardiovascular triggering during the acquisition process.

Pulsatile brain tissue displacements, induced by cardiovascular pulsation and referred to here as nonrigid motions, result from the dynamics of blood and CSF flow during the cardiac cycle (Greitz et al., 1992; Poncelet et al., 1992). In humans, the largest movements occur during the systolic period of the cardiac cycle, mostly impacting the brainstem, with a peak velocity of the parenchyma in these regions reaching ~2 mm/s in the cranio-caudal direction. During this period, thalamic tissues are also compressed at velocities inferior to 1.5 mm/s in the cranio-caudal and medio-lateral directions (Poncelet et al., 1992). In the diastolic period, the brain recovers its initial form slowly. We conducted a pilot study to determine the optimal delay between the R-wave of ECG and the trigger pulse. This showed that nonlinear phase-errors are at their minimum amplitudes when data are collected either at the early beginning of the cardiac cycle (Trigger delay = 0 s) or during the end-diastolic period of the cardiac cycle (Trigger delay = 70-90% of the R-R interval) (see Supplementary Data S8). This range of optimal trigger delay is consistent with previous human studies (Nunes et al., 2005).

This study shows that elastic pulsatile deformations of macaque brain tissue introduce inter-shot inconsistencies to DW-images collected using non-triggered 3D-msEPI, which lead to phase-errors with nonlinear spatial dependence on the phase-maps (Fig. 1A and C). These phase-errors corrupt DW-images via ghosting artifacts and by MR-signal loss mainly located in the brainstem, cerebellum, and thalamus (Fig. 1B and D).

The impact of inter-shot phase-errors on DW-images is variable and depends on the diffusion encoding direction, reaching its maximum when diffusion gradient is applied along the inferior-superior orientation of the macaque brain (Nunes et al., 2005). This results in a blurring effect on DTI-maps and a loss of anatomical details, particularly in the brainstem (Fig. 3), thalamus (Fig. 5), and cerebellum (Fig. 6), even at high resolution. Here, we have demonstrated the value of cardiovascular triggering in improving the sharpness of DTI-maps and recovering its ability to describe fine brain structure (Fig. 3). Interestingly, cardiovascular pulsations could affect the tractography of white matter bundles that cross the brainstem, the cerebellum, and the thalamus. For example, a mis-estimation of the diffusion tensor orientation was observed in the cerebellum region when non-triggered dMRI was used (Fig. 6). This leads to an increase in the false-positive and the true-negative tracts of the medial cerebellum peduncle and the inferior cerebellar peduncle (Fig. 7). No visual difference between triggered and non-triggered diffusion-tensor maps was observed in other regions of the brain that are less affected by elastic tissue deformation.

Quantitatively, the SNR measurements performed on the 8 triggered and the 8 non-triggered DW-images collected at a spatial-resolution of 0.5 mm and with b = 1500 s/mm^2^ reveal significant signal dropout of WM and GM localized only in the brainstem, cerebellum, and thalamus when the dMRI was not triggered (Supplementary Data S4, S6, and S7). Cardiovascular triggering recovered the signal dropout and also reduced the mean GSR value from 16.5 ± 10% to 4.7 ± 4.2% (Supplementary Data S5). The slight residual ghosts detected on triggered DW-images could be induced by mechanical vibrations generated in the MRI scanner during the data acquisition, by B0-inhomogeneities, and/or by Eddy currents (Alhamud et al., 2016). We have observed such residual ghosts in phantom (Kiwi) images obtained using the 3D-msEPI (GSR of 4.6 ± 1.8%).

The resolution achieved in this study (0.064 mm³) enables the visualization and exploration of very fine substructures of the brain, such as those of the brainstem presented in Figure 3, which have until now only been detectable post-mortem using dMRI (Saleem et al., 2021). These fine substructures become less and less visible as the *in-vivo* resolution decreases (Fig. 4). It should be noted that standard DTI maps, obtained using the conventional 2D-ssEPI method with standard coil and MR scanner, exhibit not only lower spatial-resolution, but also a much lower SNR than the down-sampled maps shown in Figure 4, which further reduces its ability to capture fine brain structure.

Although the cardiovascular triggering of 3D-msEPI provides a clear improvement in the quality of DW-images, it also presents certain drawbacks. First, the cardiovascular triggering forces the TR to be a multiple of the R-R interval, which may increase the total acquisition time. Second, the R-R interval must be stable over the relatively long acquisition time. In this study, both the isoflurane concentration and animals’ temperature were regularly adjusted to maintain a constant R-R interval throughout the dMRI scans (Table 2). The mean standard deviation of the heart rate for all the triggered acquisitions achieved in this study is 1.4%. Two macaques, which exhibited episodic cardiac arrhythmias disrupting the dMRI cardiac gating, were excluded from the study.

The long acquisition time is the major drawback of the triggered 3D-msEPI dMRI. In this study, the acquisition of dMRI data with 23 DW-images at the isotropic resolution of 0.4 mm required two hours, which commonly corresponds to the whole duration of an anesthetized macaque brain MRI session. Therefore, when very high-resolution dMRI data are required, structural MRI and possibly resting-state functional MRI should be collected in a separate session if necessary.

Various strategies could be employed to reduce the 3D-msEPI dMRI duration. In contrast to 2D-EPI, parallel imaging techniques can reduce 3D-EPI sampling of the Fourier-space simultaneously in both *k_y_* and *k_z_* directions, which could lead to an acceleration factor up to 10-fold (2-fold in *k_y_* and 5-fold in *k_z_*) (Narsude et al., 2016). It should be noted that the RF coil setup used in this study prevents any acquisition acceleration by parallel imaging.

Further acceleration of 3D-msEPI acquisition could be achieved by combining parallel imaging with other techniques, such as the multi-slab method (Wu et al., 2016), zoomed field-of-view (Gaggl et al., 2014; Jeong et al., 2005), and/or simultaneous multi-slice (Setsompop et al., 2018). Despite its potential to be accelerated, the ability of the 3D-msEPI sequence to achieve *in-vivo* multi-shell MRI with high angular resolution remains relatively restricted. It can thus be recommended when very high spatial-resolution and/or high diffusion weighting is required, typically in order to analyze brain microstructure using models such as DTI or NODDI (neurite orientation dispersion and density imaging).

## 5 Conclusion

Cardiovascular pulsations induce intershot phase-errors with a nonlinear spatial distribution on DW-images of anesthetized macaque brains. These phase-errors are associated with MR-signal losses as well as with ghosting artifacts on the DW-images and deteriorations of the DTI maps and of the tractography results, particularly in the brainstem, thalamus and cerebellum regions.

This study demonstrates the value of triggered 3D-msEPI in addressing cardiovascular pulsation effects and generating *in-vivo* DW-images of the macaque brain with a high spatial-resolution (up to 400 microns).

## Supplementary Material

Supplementary Material

## Data Availability

The datasets can be made available from the corresponding author on reasonable request.
