# Supplementary material for: Cardiovascular effects on high-resolution 3D multi-shot diffusion MRI of the rhesus macaque brain
Source: Imaging Neurosci (Camb). 2023 Dec 8;1:imag-1-00039. doi: 10.1162/imag_a_00039 (PMC12007547; doi:10.1162/imag_a_00039)
Supplement: Supplementary Material [file imag_a_00039-supp.pdf]

Supplementary Data

Supplementary Data S1. Ex-vivo and sub-millimetric in-vivo dMRI studies of macaque brain.

Reference	Resolution (mm)	Diffusion directions	B-value (s/mm^2)	Acquisition time/image	B0-field (Tesla)	Study type
(D'Arceuil et al., 2007)	0.425	20	4025	1 h 15 min	4.7	Ex-vivo
(Wedeen et al., 2008)	0.512	515	Up to 40000	-	4.7	Ex-vivo
(Calabrese et al., 2014)	0.4	120	4000	–	7	Ex-vivo
(Thomas et al., 2014)	0.25	121	4800	35.2 min	7	Ex-vivo
(Reveley et al., 2015)	0.25	126	4800	29 min	7	Ex-vivo
(Calabrese et al., 2015)	0.15	20	1500	2 h 18 min	7	Ex-vivo
(Azadbakht et al., 2015)	0.43	120	8000	13.5 min	4.7	Ex-vivo
(Donahue et al., 2016)	0.43	120	8000	13.5 min	4.7	Ex-vivo
(Catani et al., 2017)	0.5	61	4310	_	4.7	Ex-vivo
(Schilling et al., 2019)	0.3	31	1200	1 h 36.8 min	9.4	Ex-vivo
(Janssens et al., 2012)	0.7	256	1000	0.7 min	3	In-vivo
(Tounekti et al., 2018)	0.5	30	1000	4.4 min	3	In-vivo
(Saleem et al., 2021)	0.2	112	Up to 7000	50 min	7	Ex-vivo
(Grier et al., 2022)	0.58	127	Up to 2000	1.28 min	10.5	In-vivo
Current study	0.4	22	1000	5.45 min	3	In-vivo

Supplementary Data S2. Triggering schema used with the 3D multishot EPI diffusion MRI pulse sequence. Each EPI shot is gated in the end-diastolic phase of the cardiac cycle, at a delay corresponding to 70% of the R-R interval (green). The acquisition was triggered once every two cardiac cycles.

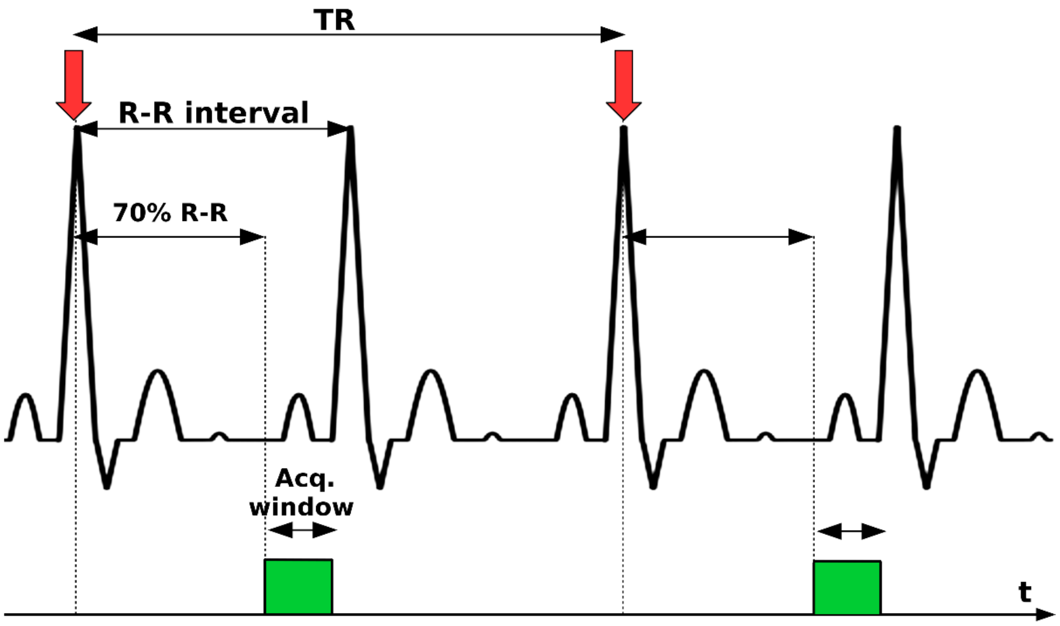

Supplementary Data S3. The diffusion direction vectors used for the DTI acquisition (Table 2).

Diffusion vector	X	Y	Z
1	0.061	0.958	0.281
2	-0.989	0.096	-0.114
3	-0.132	-0.272	0.953
4	-0.616	0.448	0.648
5	-0.647	-0.711	0.276
6	-0.569	-0.182	-0.802
7	-0.453	0.788	-0.417
8	-0.023	-0.54	-0.841
9	0.826	0.183	-0.533
10	0.096	0.85	-0.518
11	0.754	0.554	0.353
12	0.714	-0.694	-0.088
13	0.523	-0.356	0.775
14	0.409	-0.095	-0.908
15	0.451	0.883	0.131
16	-0.836	0.375	-0.402
17	0.352	-0.827	-0.438
18	0.468	0.526	-0.71
19	0.948	0.315	-0.038
20	0.172	0.098	0.98
21	0.181	-0.977	0.109

Supplementary Data S4. *Differences in SNR between triggered and untriggered dMRI acquisitions for two regions of interest manually delineated in the cerebellum and the brainstem. *** denotes p-values <0.0001.*

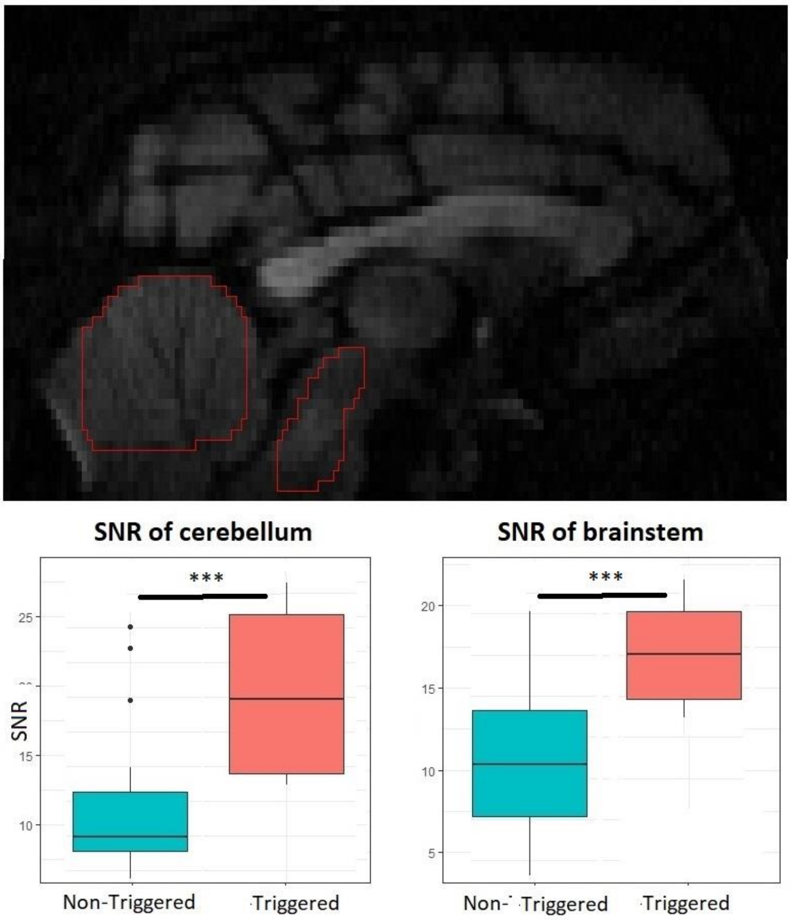

Supplementary Data S5. The region of interests (ROIs) used to assess the ghost to signal ratio (GSR). The ghost-ROI is defined on non-triggered DW-images to surround the strongest ghost outside the head, while the signal-ROI is shaped around the corpus callosum on triggered images. For each ROI, the median, first and third quartiles, and 95% confidence interval of the median are given for GSR assessed from the 8 triggered and 8 untriggered DW-images. *** denotes *p*-values <0.0001.

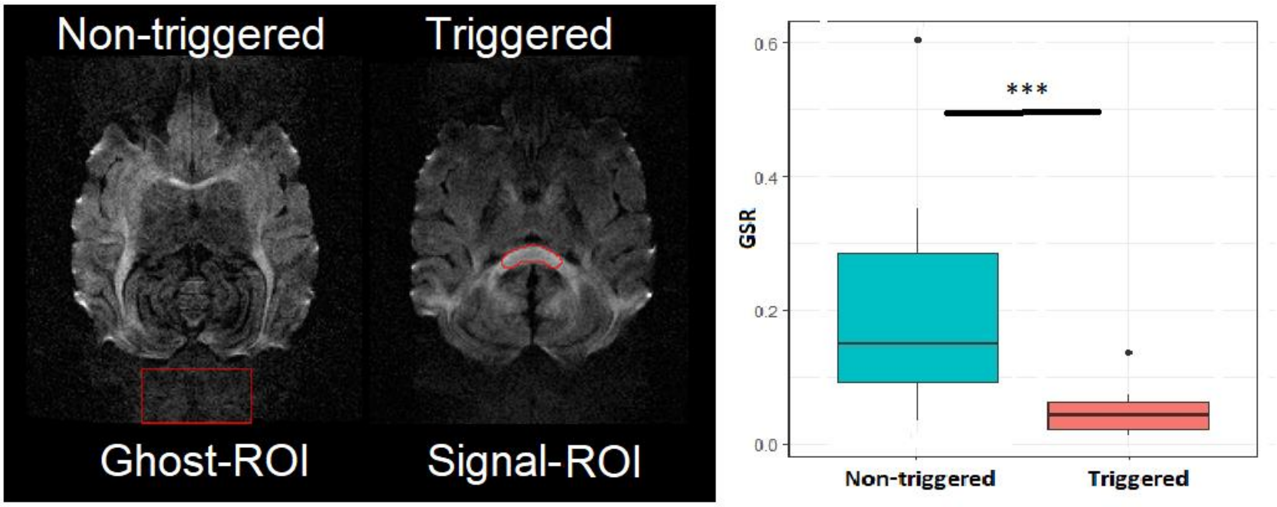

***Supplementary Data S5*: Differences in signal to noise ratio (SNR) between the triggered and non-triggered condition for white-matter ROI.** For each white-matter ROI, the median, first and third quartiles, and 95% confidence interval of the median are given for SNR assessed from the 8 triggered and 8 untriggered DW-images. Macaque brain images were acquired with a b=1500 s/mm² and with a spatial resolution of 0.5 mm for the following ROIs: splenium of the corpus callosum (SCC), genu of the corpus callosum (GCC), anterior commissure (AC), cingulum (CG), and posterior thalamic radiation (PTR). The hemispheric position is indicated by l (left), r (right) and c (center). *** denotes p-values <0.0001.

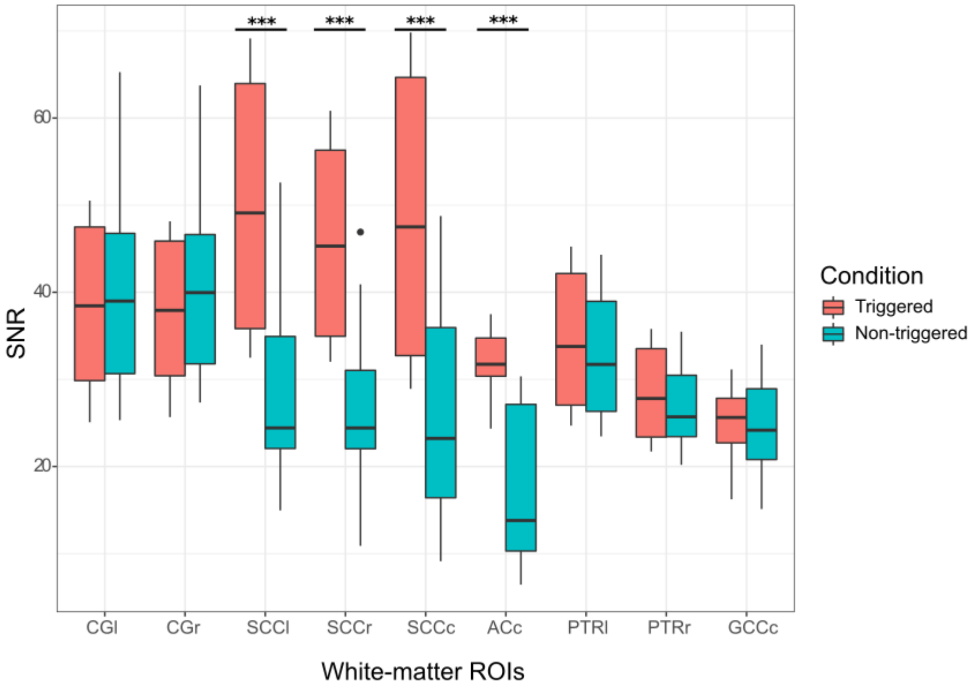

Supplementary Data S6. Differences in SNR between the triggered and non-triggered condition for each gray-matter ROI. For each gray-matter ROI, the median, first and third quartiles, and 95% confidence interval of the median are given for SNR assessed from the 8 triggered and 8 untriggered DW-images. Macaque brain images were acquired with a b=1500 s/mm² and with a spatial resolution of 0.5 mm for the following ROIs: thalamus (TH), supramarginal gyrus (SMG), precentral gyrus (PG), superior temporal gyrus (STG) and middle temporal gyrus (MTG). The hemispheric position is indicated by l (left) and r (right). *** denotes *p*-values <0.0001.

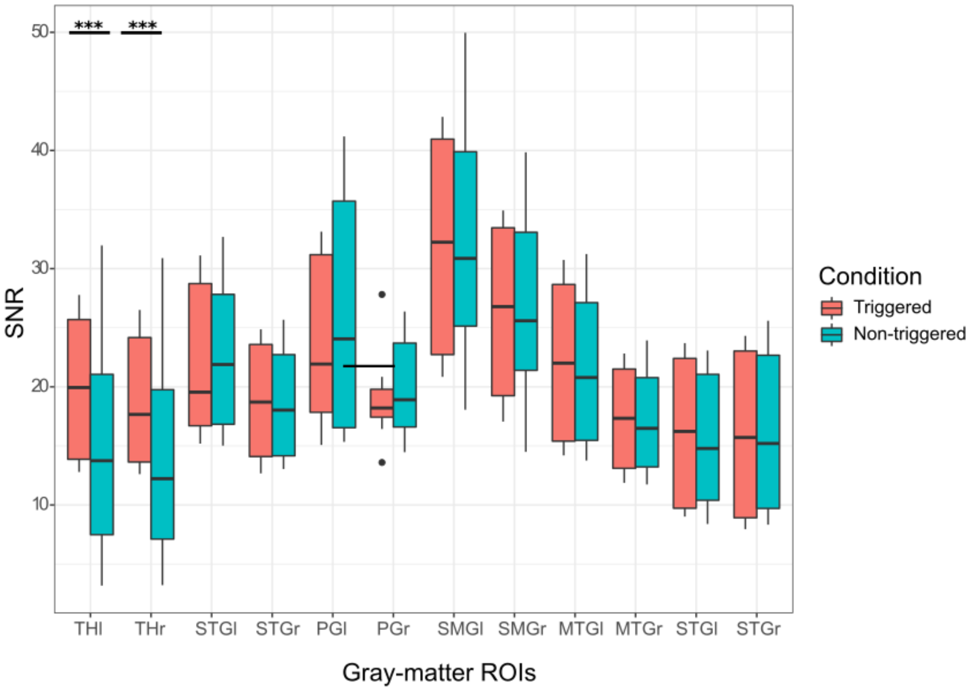

Supplementary Data S8. A. The phase maps of the additional DW-images, collected using the Acq.1 protocol without triggering (NT), and with the trigger delay set at T=0%, 30%, 50%, 70% and 90% of the R-R interval, respectively. These show the drastic decrease in nonlinear phase errors produced thanks to cardiovascular triggering. B. Phase curves plotted with the red line, defined throughout the brain on the non-triggered phase map. C. The standard deviations computed for the phase curves plotted in B and averaged over the 8 repetitions, showing that the nonlinear phase errors reach minimum values with a trigger delay of 0%, 70% or 90% of the R-R interval.

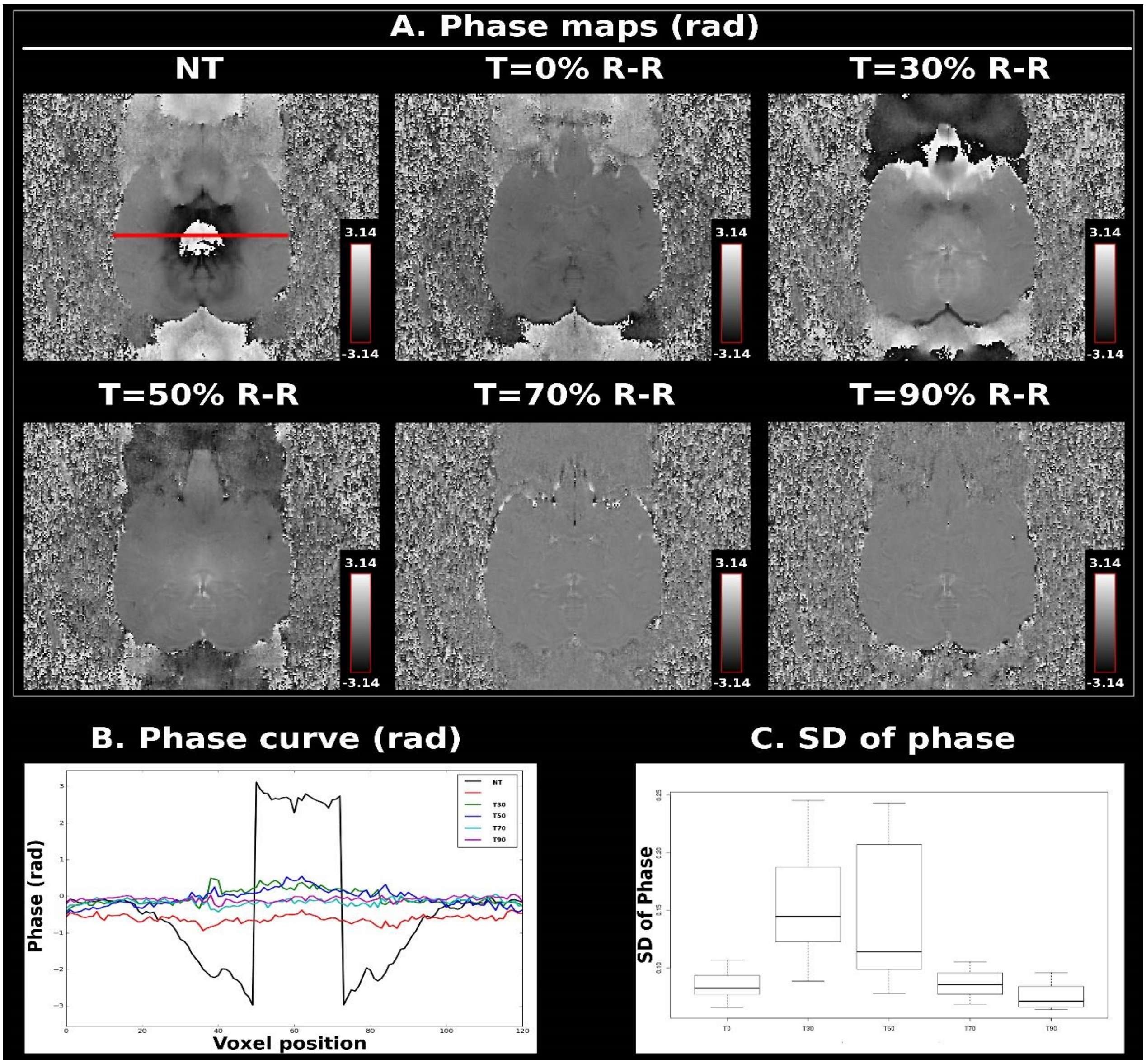

Supplementary Data References

Azadbakht, H., Parkes, L. M., Haroon, H. A., Augath, M., Logothetis, N. K., de Crespigny, A., D'Arceuil, H. E., & Parker, G. J. M. (2015). Validation of High-Resolution Tractography Against In Vivo Tracing in the Macaque Visual Cortex. *Cerebral Cortex (New York, NY)*, *25*(11), 4299-4309. https://doi.org/10.1093/cercor/bhu326

Calabrese, E., Badea, A., Coe, C. L., Lubach, G. R., Shi, Y., Styner, M. A., & Johnson, G. A. (2015). A diffusion tensor MRI atlas of the postmortem rhesus macaque brain. *NeuroImage*, *117*, 408-416. https://doi.org/10.1016/j.neuroimage.2015.05.072

Calabrese, E., Badea, A., Coe, C. L., Lubach, G. R., Styner, M. A., & Johnson, G. A. (2014). Investigating the tradeoffs between spatial resolution and diffusion sampling for brain mapping with diffusion tractography : Time well spent? *Human Brain Mapping*, *35*(11), 5667-5685. https://doi.org/10.1002/hbm.22578

Catani, M., Robertsson, N., Beyh, A., Huynh, V., de Santiago Requejo, F., Howells, H., Barrett, R. L. C., Aiello, M., Cavaliere, C., Dyrby, T. B., Krug, K., Ptito, M., D'Arceuil, H., Forkel, S. J., & Dell'Acqua, F. (2017). Short parietal lobe connections of the human and monkey brain. *Cortex; a Journal Devoted to the Study of the Nervous System and Behavior*, *97*, 339-357. https://doi.org/10.1016/j.cortex.2017.10.022

D'Arceuil, H. E., Westmoreland, S., & de Crespigny, A. J. (2007). An approach to high resolution diffusion tensor imaging in fixed primate brain. *NeuroImage*, *35*(2), 553-565. https://doi.org/10.1016/j.neuroimage.2006.12.028

Donahue, C. J., Sotiropoulos, S. N., Jbabdi, S., Hernandez-Fernandez, M., Behrens, T. E., Dyrby, T. B., Coalson, T., Kennedy, H., Knoblauch, K., Van Essen, D. C., & Glasser, M. F. (2016). Using Diffusion Tractography to Predict Cortical Connection Strength and Distance : A Quantitative Comparison with Tracers in the Monkey. *The Journal of Neuroscience: The Official Journal of the Society for Neuroscience*, *36*(25), 6758-6770. https://doi.org/10.1523/JNEUROSCI.0493-16.2016

Grier, M. D., Yacoub, E., Adriany, G., Lagore, R. L., Harel, N., Zhang, R.-Y., Lenglet, C., Uğurbil, K., Zimmermann, J., & Heilbronner, S. R. (2022). Ultra-high field (10.5T) diffusion-weighted MRI of the macaque brain. *NeuroImage*, *255*, 119200. https://doi.org/10.1016/j.neuroimage.2022.119200

Janssens, T., Keil, B., Farivar, R., McNab, J. A., Polimeni, J. R., Gerits, A., Arsenault, J. T., Wald, L. L., & Vanduffel, W. (2012). An implanted 8-channel array coil for high-resolution macaque MRI at 3T. *NeuroImage*, *62*(3), 1529-1536. https://doi.org/10.1016/j.neuroimage.2012.05.028

Reveley, C., Seth, A. K., Pierpaoli, C., Silva, A. C., Yu, D., Saunders, R. C., Leopold, D. A., & Ye, F. Q. (2015). Superficial white matter fiber systems impede detection of long-range cortical connections in diffusion MR tractography. *Proceedings of the National Academy of Sciences of the United States of America*, *112*(21), E2820-2828. https://doi.org/10.1073/pnas.1418198112

Saleem, K. S., Avram, A. V., Glen, D., Yen, C. C.-C., Ye, F. Q., Komlosh, M., & Basser, P. J. (2021). High-resolution mapping and digital atlas of subcortical regions in the macaque monkey based on matched MAP-MRI and histology. *NeuroImage*, *245*, 118759. https://doi.org/10.1016/j.neuroimage.2021.118759

Schilling, K. G., Nath, V., Hansen, C., Parvathaneni, P., Blaber, J., Gao, Y., Neher, P., Aydogan, D. B., Shi, Y., Ocampo-Pineda, M., Schiavi, S., Daducci, A., Girard, G., Barakovic, M., Rafael-Patino, J., Romascano, D., Rensonnet, G., Pizzolato, M., Bates, A., … Landman, B. A. (2019). Limits to anatomical accuracy of diffusion tractography using modern approaches. *NeuroImage*, *185*, 1-11. https://doi.org/10.1016/j.neuroimage.2018.10.029

Thomas, C., Ye, F. Q., Irfanoglu, M. O., Modi, P., Saleem, K. S., Leopold, D. A., & Pierpaoli, C. (2014). Anatomical accuracy of brain connections derived from diffusion MRI tractography is inherently limited. *Proceedings of the National Academy of Sciences of the United States of America*, *111*(46), 16574-16579. https://doi.org/10.1073/pnas.1405672111

Tounekti, S., Troalen, T., Bihan-Poudec, Y., Froesel, M., Lamberton, F., Ozenne, V., Cléry, J., Richard, N., Descoteaux, M., Ben Hamed, S., & Hiba, B. (2018). High-resolution 3D diffusion tensor MRI of anesthetized rhesus macaque brain at 3T. *NeuroImage*, *181*, 149-161. https://doi.org/10.1016/j.neuroimage.2018.06.045

Wedeen, V. J., Wang, R. P., Schmahmann, J. D., Benner, T., Tseng, W. Y. I., Dai, G., Pandya, D. N., Hagmann, P., D'Arceuil, H., & de Crespigny, A. J. (2008). Diffusion spectrum magnetic resonance imaging (DSI) tractography of crossing fibers. *NeuroImage*, *41*(4), 1267-1277. https://doi.org/10.1016/j.neuroimage.2008.03.036
